# Deconstructing the relationships between self‐esteem and paranoia in early psychosis: an experience sampling study

**DOI:** 10.1111/bjc.12263

**Published:** 2020-08-30

**Authors:** Manel Monsonet, Thomas R. Kwapil, Neus Barrantes‐Vidal

**Affiliations:** ^1^ Departament de Psicologia Clínica i de la Salut Universitat Autònoma de Barcelona Barcelona Spain; ^2^ Department of Psychology University of Illinois at Urbana‐Champaign USA; ^3^ Sant Pere Claver – Fundació Sanitària Barcelona Spain; ^4^ Centre for Biomedical Research Network on Mental Health (CIBERSAM) Instituto de Salud Carlos III Barcelona Spain

**Keywords:** Paranoia, self‐esteem, early psychosis, experience sampling, social support

## Abstract

**Background:**

No studies have examined the association between self‐esteem and paranoia developmentally across the critical stages of psychosis emergence. The present study fills this gap and extends previous research by examining how different dimensions, measures, and types of self‐esteem relate to daily‐life paranoia across at‐risk mental states for psychosis (ARMS) and first episode of psychosis (FEP) stages. Furthermore, the moderation effects of momentary anxiety and momentary perceived social support on the association between momentary self‐esteem and paranoia were examined.

**Design:**

This study used a multilevel, cross‐sectional design.

**Methods:**

One‐hundred and thirteen participants (74 ARMS and 39 FEP) were assessed repeatedly over seven consecutive days on levels of momentary paranoia, self‐esteem, anxiety and perceived social support using experience sampling methodology. Measures of trait and implicit self‐esteem were also collected.

**Results:**

Global momentary and trait self‐esteem, and their positive and negative dimensions, were related to daily‐life paranoia in both ARMS and FEP groups. Conversely, implicit self‐esteem was not associated with daily‐life paranoia in either group. Anxiety negatively moderated the association between positive self‐esteem and lower paranoia, whereas both feeling close to others and feeling cared for by others strengthened this association. However, only feeling cared for by others moderated the association between negative self‐esteem and higher paranoia.

**Conclusions:**

Different types, measures and dimensions of self‐esteem are differentially related to paranoia in early psychosis and are influenced by contextual factors in daily‐life. This yields a more complex picture of these associations and offers insights that might aid psychological interventions.

**Practitioner points:**

- Different measures (trait and momentary) and dimensions (positive and negative) of explicit self‐esteem are distinctly related to paranoia across risk and first‐episode stages of psychosis.
- Explicit, but not implicit, self‐esteem is associated with real‐life paranoia in incipient psychosis.
- Anxiety boosted the association of poor self‐esteem and paranoia ideation in daily‐life.
- Social closeness, but feeling cared for by others in particular, interacts with self‐esteem tempering the expression of paranoia in real life.

Paranoia is a prominent positive symptom in psychotic disorders, the psychosis prodrome and subclinical schizotypy (e.g. Horton, Barrantes‐Vidal, Silva, & Kwapil, 2014). In individuals with a first episode of psychosis (FEP), rates of persecutory delusions range from over 70% (Coid *et al*., 2013) to 90% (Tarrier *et al*., 2004). Likewise, in the prodromal phase for psychosis, paranoia was among the most prevalent symptoms (Lencz, Smith, Auther, Correll, & Cornblatt, 2004; Salokangas *et al*., 2016; Zhang *et al*., 2014) and strongly related to transition to psychosis (Cannon *et al*., 2008). Examining the aetiological underpinnings of paranoia in the early stages of psychosis should provide both complementary and clearer information than that obtained from the more developed stages of the disorder (Kwapil & Barrantes‐Vidal, 2015), as such studies avoid many of the confounding effects generated by demoralization, stigma, long‐term medication, chronicity and comorbid characteristic of chronic psychosis. Furthermore, comparing persons with at‐risk metal states for psychosis (ARMS) versus FEP should facilitate our ability to distinguish aetiologically relevant onset mechanisms from consequences of psychotic disorders, and better understand the role of persecutory ideation in the development of psychotic disorders.

Both implicit (ISE) and explicit (ESE) self‐esteem are implicated as causal factors in the development of paranoia. Whereas ESE is characterized by a reflective and conscious attitude towards oneself, ISE involves a more automatic or unconscious self‐evaluation. Bentall, Corcoran, Howard, Blackwood and Kinderman (2001) proposed a model based on a cycle of mutual influences between causal attributions and self‐representations, suggesting that paranoia can serve as a defence against low ISE. They predicted that people with persecutory delusions would have low ISE, measured by reaction time tasks, and that a discrepancy between ISE and ESE would be expected, being ESE higher than ISE. By contrast, Freeman, Garety, Kupiers, Fowler & Bebbington (2002) proposed a model in which paranoia is conceptualized as threat anticipation beliefs and highlight the direct role of negative emotions, particularly anxiety, in the formation and maintenance of paranoia ideation. The model regards low ESE as critical component of putative multifactorial pathways that can lead to the development of paranoia. Recent reviews (Kesting & Lincoln, 2013; Murphy, Bentall, Freeman, O'Rourke, & Hutton, 2018; Tiernan, Tracey, & Shannon, 2014) concluded that impaired ESE is associated with paranoia in clinical and non‐clinical groups, whereas the association between low ISE and paranoia remains equivocal. Further research is needed to explore self‐esteem beyond its global conceptualization to disentangle the specific pathways of delusion formation. For example, positive and negative self‐evaluations show differential connections with paranoia (Palmier‐Claus, Dunn, Drake, & Lewis, 2011a; Stewart *et al*., 2017). However, whereas most of studies explored negative and positive self‐schemas (e.g. Fowler *et al*., 2012; Freeman *et al*., 2008; Smith *et al*., 2006), only a few assessed the positive and the negative dimensions of ESE (e.g. Bentall *et al*., 2008; Udachina *et al*., 2009).

ESE is traditionally viewed as a bipolar construct with positive and negative dimensions placed at opposite poles (Marsh, 1986; Rosenberg, 1965). However, it is possible to hold both intense and self‐contradictory feelings about oneself (Higgins, 1987). In fact, 'splitting the self' into separate positive and negative selves could be a defensive mechanism for dealing with negative experiences and self‐conceptions, and facilitating emotional change (Bowlby, 1980; Chadwick, 2003; Grennberg, Rice, & Elliott, 1993; Sullivan, 1953). Thus, alternative formulations characterize ESE as separate dimensions of positive and negative self‐esteem (Andrews, 1998; Brown *et al*., 1990; Khon & Schooler, 1969; Owens, 1993; Shahani, Dipboye, & Phillips, 1990), similar to the conceptualization of affect as two separate but correlated factors of positive and negative affect (Tellegen, Watson, & Clark, 1999; Watson & Clark, 1984). Indeed, Barrowclough *et al*. (2003) asserted that positive and negative ESE not only make independent contributions to global ESE, but also to affect and behaviour.

The Rosenberg Self‐Esteem Scale (RSES; Rosenberg, 1979) is the most widely used questionnaire of global ESE, although there is on‐going debate as to whether it should be treated as a unidimensional scale (e.g. Marsh, Scalas, & Nagengast, 2010; Tomas & Oliver, 1999) or separate positive and negative factors (e.g. Kaufman, Rasinski, Lee, & West, 1991 *;* Owens, Stryker, & Goodman, 2001). Huang and Dong's (2012) meta‐analysis reported that a 2‐factor structure was supported, but recommended using a 1‐factor solution unless positive and negative factors showed distinct associations with other important constructs. Only one study used the RSES to explore its negative and positive dimensions in relation to paranoia (Palmier‐Claus *et al*., 2011a), showing that paranoia was differentially associated with negative and positive ESE in FEP patients. However, there are no studies examining this distinction comparing ARMS and FEP participants.

Despite the fact that associations between self‐esteem and paranoia might occur on a momentary basis in the real world, studies of these constructs often rely on one‐time retrospective reports in laboratory or clinical settings. Experience sampling methodology (ESM) is a structured diary technique that assesses cognition, affect, symptoms and contextual factors in daily‐life (Myin‐Germeys et al., 2003). ESM offers advantages to traditional cross‐sectional procedures, as it: (a) assesses mental experiences in their natural context, increasing ecological validity; (b) explores the interaction of the individual with the environment; and (c) decreases retrospective bias. Previous ESM studies with psychotic and non‐clinical participants found that global low ESE was associated with paranoia when examined concurrently and with time‐lagged analysis (Palmier‐Claus, Dunn, Morrison, & Lewis, 2011b; Thewissen, Bentall, Lecomte, van Os, & Myin‐Germeys, 2008; Thewissen *et al*., 2011; Udachina, Varese, Myin‐Germeys, & Bentall, 2014). However, one study that explored the association of momentary positive and negative ESE with paranoia did not observe a temporal association of ESE with paranoia in non‐clinical participants (Udachina *et al*., 2009). Finally, to our knowledge, there are no studies investigating *momentary* moderators of the relationship between ESE and paranoia.

As anxiety has been proposed (Freeman *et al*., 2002) and validated (Ben‐Zeev, Ellington, Swendsen, & Granholm, 2011; Thewissen *et al*., 2011) as a predictor of the occurrence of persecutory ideation, it seems plausible that the mutual influence of momentary anxiety and low self‐esteem could have a detrimental impact on momentary levels of paranoia. By contrast, given the relevance of perceived social support for improving psychotic symptoms in FEP patients (Norman *et al*., 2005; Sündermann, Onwumere, Kane, Morgan, & Kuipers, 2014), the interaction of momentary perceived social support and ESE might diminish paranoia. In fact, paranoia has been associated with low perceived social support (Freeman *et al*., 2011) and reduced social networks and support seems to pre‐date onset of psychotic disorder (Gayer‐Anderson & Morgan, 2013).

Different methods of assessment (i.e. ESM, trait, and retrospective measures) reflect different nuances of subjective experience with different implications for research and clinical practice (Delespaul, 1995). Thus, using ESM or trait questionnaires to assess mental phenomena might capture distinct but complementary information (Conner & Barret, 2012). Whereas ESM taps ESE in the present moment (based primarily on contextual factors and current experiences), trait measures capture a more reflective ESE by activating long‐term experiences and memories. Previous studies have showed the convergent validity of trait and ESM measures of ESE (Udachina *et al*., 2009; Udachina, Varese, Oorschot, Myin‐Germeys, & Bentall, 2012), although some subtle distinctions appeared.

The association between different dimensions (global, positive and negative) and measures of ESE with momentary paranoia are understudied, and the association between ISE and momentary paranoia has not been examined. Moreover, to our knowledge, no previous studies have explored these associations across risk and first‐episode stages. Therefore, the first aim of this study was to examine whether global and specific (positive and negative) dimensions of ESE show differential associations with momentary paranoia. Additionally, we examined whether such associations hold for *momentary* self‐reports (ESM) and *trait* (RSES) ESE. Based on previous findings, negative associations of global momentary and trait ESE with paranoia were expected. Given the paucity of previous studies, the analyses about positive and negative dimensions of ESE are exploratory. Second, we tested whether the effects of global, positive, and negative momentary and trait ESE on paranoia differed between ARMS and FEP stages. As ARMS participants have not reached the psychosis threshold, we predicted stronger associations of global and positive momentary ESE with lower levels of paranoia in the ARMS group. Third, we explored whether ISE was related to momentary paranoia, and whether this association varied across ARMS and FEP. Finally, we examined whether momentary anxiety and perceived social support moderated the association between dimensions of ESE and paranoia in daily‐life, and whether such moderation effects differed between ARMS and FEP. We hypothesized that anxiety would strengthen the association between poor ESE and paranoia, whereas positive interpersonal appraisals would temper this association in both groups.

## Method

### Participants and procedure

The present study is part of a larger longitudinal study at four Public Mental Health Centres in the Sant Pere Claver‐Early Psychosis Program (SPC‐EPP; Domínguez‐Martínez *et al*., 2014) in Barcelona. We invited 164 (97 ARMS and 67 FEP) individuals to collaborate in the study, of whom 36 (17 ARMS and 19 FEP) refused to collaborate. The initial sample consisted of 128 (80 ARMS and 48 FEP) participants. However, 6 ARMS and 9 FEP participants were excluded from the analyses due to missing data on the self‐esteem measures or invalid ESM protocols. Thus, the final sample of this study included 74 ARMS and 39 FEP participants (mean age = 22.5 years, *SD* = 4.6 years; 68.5 % males). ARMS criteria were based on the Comprehensive Assessment of At‐Risk Mental States (CAARMS; Yung *et al*., 2005). None of the ARMS patients met DSM‐IV‐TR (American Psychiatric Association, 2000) criteria for any psychotic disorder or affective disorder with psychotic symptoms as assessed by the Structured Clinical Interview for DSM‐IV (SCID‐I; First, Spitzer, Gibbon, & Williams, 1995). FEP patients met DSM‐IV‐TR criteria for any psychotic disorder or affective disorder with psychotic symptoms assessed by the SCID‐I for DSM‐IV. Patients’ inclusion criteria were age between 14 and 40 years old and IQ ≥ 75. Exclusion criteria were evidence of organically based psychosis and any previous psychotic episode. All participants provided informed consent and completed the assessment protocol within maximum of 4 weeks. The project was developed after the Code of Ethics of the World Medical Association (Declaration of Helsinki) and was approved by the local ethical committee.

### Measures

#### ESM measures

Participants received a personal digital assistant (*n* = 72) or a smartphone (*n* = 41) that signalled randomly 8 times daily (between 11 am and 10 pm) for seven days to complete brief questionnaires. Participants employing smartphones were signalled via text message to complete the ESM questionnaire online using Qualtrics survey software. The average of completed ESM questionnaires was identical for the personal digital assistant (35.3; range: 18–56) and smartphones (35.5; range: 18–56). Studies indicate that these methods produce similar data in terms of quantity and quality (Burgin, Silvia, Eddington, & Kwapil, 2013; Kimhy, Myin‐Germeys, Palmier‐Claus, & Swendsen, 2012). Participants had 15 minutes after the signal to complete the questionnaire. After Delespaul (1995), 3 participants who had less than a third (18) valid questionnaires at the end of the assessment were excluded from the analysis. The English translation of the complete ESM questionnaire can be found in supplementary material (Table S1). A detailed description of the ESM assessment and validation data can be found in previous studies (Barrantes‐Vidal, Chun, Myin‐Germeys, & Kwapil, 2013; Kwapil, Brown, Silvia, Myin‐Germeys, & Barrantes‐Vidal, 2012). Items were rated on a 7‐point Likert scale that ranges from 'not at all' to 'very much'. Several studies have employed ESM across the psychosis continuum showing its validity and reliability (Barrantes‐Vidal *et al*., 2013; Kwapil *et al*., 2012; Reininghaus *et al*., 2016; Thewissen *et al*., 2011). Within‐ and between‐person reliabilities for ESM indices were computed after Geldhof, Preacher and Zyphur (2014). Global momentary ESE was measured with the mean of 3 ESM items ('Right now I feel good about myself', 'Right now I can cope' and 'Right now I feel guilty or ashamed', reversed; within alpha = .51, between alpha = .83). The first two items comprise the momentary positive ESE index (within alpha = .49, between alpha = .94), and the latter item assesses momentary negative ESE.

Momentary paranoia was assessed with the mean of 2 ESM items ('Right now I feel suspicious', 'Right now I feel mistreated'; within alpha = 0.53, between alpha = .83). Anxiety was measured with the mean of 3 items ('Right now I feel anxious', 'My current is situation stressful', 'Right now I feel relaxed', reversed; within alpha = 0.61, between alpha = .84). We employed two appraisals of perceived social support. One was prompted at all signals ('Right now I feel that others care about me'); the other one was prompted when participants were with others ('Right now I feel close to this person/these people'). Note that we were unable to compute an overall social support index because one of the items was only administered when participants indicated that they are with another person at the time of the signal.

### Trait self‐esteem

Trait ESE was assessed with the Spanish version of the RSES (Rosenberg, 1965). The RSES consists of 5 positively worded items and 5 negatively worded items measured on a 4‐point scale, with higher scores reflecting higher global ESE. Principal components analysis (Promax rotation) of RSES items in our sample showed a two‐factor solution (*r* = −.55). One factor represented positive ESE and the other negative ESE, accounting for 47.9% and 37.5% of the variance, respectively. Positive and negative ESE factor scores were computed for each participant.

### Implicit self‐esteem

The go/no‐go association task (GNAT; Nosek & Banaji, 2001) was employed to assess ISE. Previous studies indicate its convergent, discriminant and predictive validity (e.g. Spalding & Hardin, 1999; Teachman, 2007). The GNAT version in this study evaluated the intensity of unconscious associations between concepts of 'Self' (e.g. myself, I, participant name) and 14 positive adjectives (e.g. smart, competent) or 14 negative adjectives [e.g. unable, stupid; see Valiente *et al*. (2011)]. The GNAT had two blocks (self‐positive and self‐negative) that were randomly presented, each with 20 practice and 60 critical trails. For each trial, one word appeared in the middle of the screen, while informative labels (self and positive or self and negative) for the correct response were fixed in the upper left and right corners. Participants had to press the space bar only if the word that appeared in the middle of the screen (e.g. smart) belonged to the informative label (e.g. self and positive). Words appeared for up to 1200 ms or until the participant made a response. Participants were instructed to respond as fast and accurately as possible, and they had immediate feedback after each trial: a green *O* followed correct responses, whereas a red *X* followed incorrect responses. To calculate ISE, reaction times in the positive self‐blocks were subtracted from reaction times in the negative self‐blocks. A positive score indicated positive ISE, whereas a negative score indicated negative ISE.

### Data analyses

ESM data have a multilevel structure in which ESM ratings (level 1 data) are nested within participants (level 2 data). Level 1 predictors were group mean centred, level 2 predictors were grand mean centred, and parameter estimates were calculated using robust standard errors. Multilevel analyses were computed with MPlus 6 (Muthén & Muthén, 2010). First, a series of multilevel regressions were conducted to test the impact of different dimensions (global, positive and negative) of *momentary* ESE (level 1 predictors) on momentary paranoia. Similarly, a series of multilevel analyses were performed to explore the impact of global, positive and negative *trait* ESE, and ISE (level 2 predictors) on momentary paranoia. Second, cross‐level interactions were conducted to explore whether the effects of different dimensions (global, positive and negative) of momentary ESE on paranoia differed between ARMS and FEP groups. Cross‐level interactions tested whether level 1 slopes (the association of different dimensions of momentary ESE with paranoia) varied as a function of level 2 group variable (0 = ARMS, 1 = FEP). To explore whether the associations of ISE and dimensions of trait ESE with paranoia differed between groups, the two main effects (e.g. positive trait ESE and group) were entered at the first step, and the two‐way interaction term was entered at the second step to examine its contribution over‐and‐above the main effects. The nature of significant interactions was examined using simple slopes analyses. Third, we tested the potential moderating role of anxiety and perceived social support on the association between different dimensions of momentary ESE and paranoia in daily‐life. The two main effects (e.g. global ESE and anxiety) were entered at the first step, and the two‐way interaction terms (e.g. global ESE × anxiety) were entered at the second step. Finally, to explore whether the effects of the level 1 moderators (anxiety, and the two items of perceived social support) varied between ARMS and FEP groups, level 2 group variable was entered at the third step.

## Results

### Descriptive data for ARMS and FEP groups

Group comparisons of all variables and demographic data are presented in Table 1. There were no differences in sex composition, ethnicity, immigrant status and number of usable ESM questionnaires between groups (note we are reporting aggregate ESM values here for illustrative purposes, but subsequently analyse nested ESM data with multilevel modelling). The FEP group was significantly older, had more unoccupied individuals and showed higher global and positive trait ESE than the ARMS group. The ARMS group showed higher reports of paranoia and lower levels of momentary positive ESE and positive appraisals of others.

**Table 1:** Descriptive data and comparison of ARMS and FEP groups on study variables

	ARMS *n *= 74	FEP *n *= 39	Test statistics	
Demographics				
Age	21.56 (4.02)	24.59 (4.88)	*t *= −3.319	***p*** = **.001**
Sex (Male %)	67.6	69.2	*χ^2^* = 0.033	*p* = .857
Immigrant (%)				
No	83.8	69.2	*χ^2^* = 3.234	*p* = .072
Yes	16.2	30.8		
Ethnicity (%)				
Caucasian–white	75.7	71.8	*χ^2^* = 0.202	*p* = .653
Other	24.3	28.2		
Occupation (%)				
Unoccupied	31.5	61.5	*χ^2^* = 9.524	***p*** = **.009**
Worker/Employee	15.1	10.3		
Student	53.4	28.2		
Momentary variables^a^				
ESM usable	35.81(11.12)	34.64(10.87)	*t* = 0.536	*p* = .593
ESM Paranoia	1.92(1.10)	1.44(0.63)	*t* = 2.897	***p*** = **.005**
ESM self‐esteem	4.83(1.02)	5.21(1.11)	*t *= −1.816	*p* = .072
ESM SE positive	4.17(1.19)	4.66(1.30)	*t *= −2.475	***p*** = **.049**
ESM SE negative	1.86(1.18)	1.69(0.98)	*t* = 0.778	*p* = .438
ESM anxiety Index	2.88(0.98)	2.61(1.02)	*t* = 1.375	*p* = .172
ESM cared by others	4.05(1.57)	4.71(1.45)	*t* = −2.179	***p*** = **.031**
ESM close to others	5.05(1.43)	5.59(1.10)	*t* = −2.042	***p*** = **.044**

Trait Self‐esteem	*n *= 71	*n *= 38		
RSES Total score	14.76(6.34)	17.37(6.04)	*t* = −2.079	***p*** = **.040**
RSES positive factor	−0.17(0.95)	0.31(1.03)	*t* = −2.409	***p*** = **.018**
RSES negative factor	0.10(1.05)	−0.19(0.88)	*t* = 1.451	*p* = .150
Implicit self‐esteem (ms)	16.94(62.01)	18.92(55.10)	*t* = −0.166	*p* = .868

Note

ARMS = At‐Risk Mental State for Psychosis; FEP = First‐Episode Psychosis; ESM = Experience Sampling Method; SE = Self‐esteem; RSES = Rosenberg Self‐Esteem Scale.

^a^ Mean ESM scores for each participant were used.

### Association of self‐esteem with paranoia

Zero‐order correlations for all self‐esteem measures employed in this study are shown in Table 2. A series of multilevel regressions examined the association of global, positive and negative dimensions of momentary and trait ESE, as well as ISE, with momentary paranoia in daily‐life. As expected, both momentary and trait global ESE were inversely associated with paranoia (Tables 3, 4). Positive momentary and trait ESE were inversely related with paranoia, whereas negative momentary and trait ESE were positively associated with paranoia (Tables 3, 4). By contrast, ISE was unrelated with paranoia (0.001, *SE* = .002, *p* = .795).

**Table 2:** Zero‐order correlations of all self‐esteem variables (*n *= 113 for ESM measures and 109 for all analyses including trait measures)

	RSES total	RSES positive	RSES negative	Implicit SE	ESM SE Total ^a^	ESM SE positive ^a^
RSES total	‐‐‐					
RSES positive	.84^***^	‐‐‐				
RSES negative	−.93^***^	−.58^***^	‐‐‐			
Implicit SE	−.04	−.02	.05	‐‐‐		
ESM SE total ^a^	.58^***^	.42^***^	−.60^***^	−.11	‐‐‐	
ESM SE positive ^a^	.57^***^	.41^***^	−.58^***^	−.19^*^	.95^***^	‐‐‐
ESM SE negative ^a^	−.40^***^	−.28^**^	.42^***^	−.12	−.73^***^	−.48^***^

Note

RSES = Rosenberg Self‐esteem Scale; SE = Self‐esteem; ESM: Experience Sampling Method.

^a^ Mean ESM scores for each participant were used.

* *p* < .05, **p < .01, ***p < .001

**Table 3:** Main effects of momentary Self‐esteem and cross‐level interactions with Group status on momentary Paranoia (*n *= 113; number of observations = 4006)

Criterion	Level 1 Predictor		Level 2 Predictor
ESM Paranoia	ESM Self‐esteem		Group: ARMS vs FEP
	γ_10_ (*df* = 112)		γ_11_ (*df* = 111)
Paranoia index	Global self‐esteem	−0.342 (0.037)^***^	0.230 (0.069)^**^
Paranoia index	Positive self‐esteem	−0.245 (0.030)^***^	0.176 (0.059)^**^
Paranoia index	Negative self‐esteem	0.229 (0.024)^***^	−0.111 (0.044)^*^

Note

ESM = Experience Sampling Method; ARMS = At‐Risk Mental State for Psychosis; FEP = First‐Episode Psychosis.

* *p* < .05, **p < .01, ***p < .001.

**Table 4:** Main effects of trait Self‐esteem, Group status and their Interaction on momentary Paranoia (*n *= 109; number of observations = 3850)

Criterion	Step 1: Level 2 predictors			Step 2
ESM Paranoia	Trait self‐esteem		Group: ARMS vs FEP	Interaction term^a^
	γ_01_ (*df* = 105)		γ_02_ (*df* = 105)	γ_03_ (*df* = 104)
Paranoia index	Global self‐esteem	−0.053 (0.015)^**^	−0.299 (0.146)^*^	−0.035 (0.173)
Paranoia index	Positive self‐esteem	−0.225 (0.095)^*^	−0.331 (0.164)^*^	−0.154 (0.173)
Paranoia index	Negative self‐esteem	0.349 (0.099)^**^	−0.337 (0.167)^*^	−0.007 (0.186)

Note

ESM = Experience Sampling Method; ARMS = At‐Risk Mental State for Psychosis; FEP = First‐Episode Psychosis.

^a^ Trait Self‐esteem and Group were examined independently. The Interaction was examined with trait Self‐esteem and Group in the model.

* *p* < .050, **p < .001.

### Effect of group on the association between self‐esteem and paranoia

Multilevel regression showed that the ARMS group experienced more paranoia than the FEP group in daily‐life (−0.422, *SE* = .169, *p* = .012). Group status moderated the association of momentary global, positive and negative ESE with paranoia (Table 3). Simple slope analyses showed that all associations of momentary ESE and paranoia were significant in both groups, but were stronger in the ARMS group (momentary global ESE: −.398, *SE* = .046; momentary positive ESE: −.274, *SE* = .042; momentary negative ESE: .255, *SE* = .031; all *p’s* < .001) than in the FEP group (momentary global ESE: −.218, *SE* = .046; momentary positive ESE: −.142, *SE* = .040; momentary negative ESE: .165, *SE* = .031; all *p’s* < 0.001). Conversely, the trait ESE dimensions × group interactions were not significant, indicating that the associations of trait ESE and paranoia were invariant across ARMS and FEP groups (Table 4). Finally, the ISE × group interaction was not significant (−0.065, *SE* = .192, *p* = .734).

### Effect of level 1 moderators on associations of momentary ESE and Paranoia

Table 5 presents the interactions of potential moderators on associations of momentary global, positive and negative ESE with paranoia in the whole sample. Results showed that momentary anxiety and social closeness moderated the associations of global and positive, but not negative, ESE with paranoia. Feeling cared for by others moderated associations of all dimensions of ESE and paranoia. Thus, feeling cared for by others strengthens the association between positive or global ESE with lower levels of paranoia and attenuates the association between negative ESE with paranoia. Note that the moderation effects did not differ by group in any of the analyses.

**Table 5:** Main effects of level 1 predictors, their interaction and cross‐level interactions of Group status with level 1 interactions on Paranoia (*n *= 113)

Level 1 Criterion: ESM Paranoia						
Step 1: Level 1 Predictors				Step 2: Level 1 Interactions		Step 3: Group (ARMS vs. FEP)
γ_10_ (*df* = 111)		γ_20_ (*df* = 111)		γ_30_ (*df* = 110)		γ_31_ (*df* = 109)
Number of observations = 4006						
Self‐esteem	−0.257 (0.034)***	Anxiety index	0.185 (0.025)***	Global SE × Anxiety index	−0.042 (0.015)**	0.001 (0.029)
Positive SE	−0.162 (0.030)***	Anxiety index	0.210 (0.027)***	Positive SE × Anxiety index	−0.041 (0.013)**	−0.003 (0.027)
Negative SE	0.170 (0.021)***	Anxiety index	0.225 (0.026)***	Negative SE × Anxiety index	0.009 (0.013)	−0.009 (0.026)
Number of observations = 4006						
Self‐esteem	−0.313 (0.034)***	Cared about me	−0.073 (0.025)**	Global SE × Cared about me	0.035 (0.011)**	−0.013 (0.023)
Positive SE	−0.206 (0.029)***	Cared about me	−0.083 (0.025)**	Positive SE × Cared about me	0.023 (0.010)**	−0.019 (0.020)
Negative SE	0.211 (0.024)***	Cared about me	−0.112 (0.027)***	Negative SE × Cared about me	−0.031 (0.011)*	−0.015 (0.024)
Number of observations = 2223						
Self‐esteem	−0.250 (0.034)***	Close to others	−0.062 (0.017)***	Global SE × Close to others	0.019 (0.010)*	0.034 (0.027)
Positive SE	−0.166 (0.029)^***^	Close to others	−0.069 (0.018)^***^	Positive SE × Close to others	0.015 (0.008)^*^	0.020 (0.021)
Negative SE	0.164 (0.024)***	Close to others	−0.076 (0.016)***	Negative SE × Close to others	−0.010 (0.009)	−0.049 (0.026)

Note

ESM = Experience Sampling Method; SE = Self‐Esteem; ARMS = At‐Risk Mental State for Psychosis; FEP = First‐Episode Psychosis.

* *p* < .05, **p < .01, ***p < .001.

## Discussion

### Main findings

This study presented the first examination of how distinct measures (momentary and trait), dimensions (positive and negative) and types (explicit and implicit) of self‐esteem are related to momentary self‐reports of paranoia in ARMS and FEP samples. Additionally, the role of potential positive (e.g. feeling cared for and social closeness) and negative (anxiety) moderators on these associations was explored. Global momentary and trait ESE, as well as their positive and negative dimensions, were related to momentary paranoia. Conversely, ISE was unassociated with momentary paranoia. Additionally, ARMS and FEP patients differed in associations between all dimensions of momentary ESE and paranoia, such that these associations were stronger in the ARMS group. However, groups did not show significant differences in the association between any dimension of trait ESE and paranoia, suggesting that they tap different aspects of ESE that should be examined separately. To our knowledge, the effects of potential momentary moderators on the relationship between ESE and paranoia in daily‐life have not been previously explored. As expected, momentary anxiety strengthened the relationship between low global ESE and paranoia, whereas momentary appraisals of social closeness and feeling cared for by others tempered this association. However, the analysis of the positive and negative dimensions showed that only feeling cared for by others moderated the association between negative ESE and paranoia. These findings indicate relevant differences between positive and negative dimensions of ESE and underscore the critical role of daily‐life contextual factors in the expression of paranoia. Importantly, they highlight the power of positive social appraisals in buffering the association between poor ESE and paranoia. Specifically, feeling cared for by others, which moderated all the associations, seems to target a core component of social defeat, that is, feeling excluded by others.

### Different types, measures and dimensions of self‐esteem

Consistent with most previous research, momentary and trait global ESE were negatively associated with paranoia in ARMS and FEP groups, confirming this association occurs before the psychotic outbreak and chronic psychosis, respectively. In addition, both positive and negative momentary and trait ESE were associated with paranoia in daily‐life. However, other studies found trait negative, but not positive, ESE associated with paranoia in psychotic patients (Bentall *et al*., 2008; Palmier‐Claus *et al*., 2011a). Only a few studies separately explored positive and negative dimensions of ESE, so it is difficult to disentangle whether these associations might change as paranoia intensifies from mild ideation to delusions. As ESE (Palmier‐Claus et al., 2011b; Thewissen *et al*., 2007) and paranoia fluctuate in daily‐life (Thewissen *et al*., 2008; Udachina *et al*., 2014), and it is suggested that the relationship between ESE and paranoia changes across different stages of the disorder (Drake *et al*., 2004; Morrison *et al*., 2015), it is essential to examine the dynamic influences of positive and negative ESE on paranoia across the psychosis continuum, thereby improving our understanding of affective and cognitive mechanisms that lead to persecutory delusions.

The magnitude of the associations between dimensions of momentary ESE and paranoia were stronger in the ARMS than in the FEP group, albeit still significant in the FEP group. This confirms our hypothesis that as ARMS patients have not reached the psychosis threshold, they would experience greater beneficial effects of momentary positive ESE on paranoia. Nevertheless, ARMS patients showed a greater detrimental effect of momentary negative ESE on paranoia. This finding might seem counterintuitive, just as the fact that the ARMS group reported more momentary paranoia and lower positive ESE than the FEP group. Notwithstanding, several studies have also reported higher levels of self‐rated paranoia in ARMS as compared to FEP participants, as well as ESE and depressive symptoms (An *et al*., 2010), positive self‐schemas (Taylor *et al*., 2014), emotional reactivity to stress (Palmier‐Claus, Dunn, & Lewis, 2012) and psychotic experiences (Reininghaus *et al*., 2016; van der Steen *et al*., 2017). Of note, a recent ESM study that used previously collected data from six ESM studies in samples along the psychosis continuum (NARSAD, MAPS, EUGEI, STRIP1, STRIP2, iTHINK) found that ESM ratings of suspiciousness, tension and negative affect were higher in ARMS individuals than in chronic psychosis patients (Vaessen *et al*., 2019). Two major issues could influence this pattern of results. First, most of FEP patients are taking antipsychotic medication, whereas this is much less frequently the case in their ARMS counterparts. Second, although FEP patients meet criteria for a psychotic disorder and paranoid symptoms are highly prevalent, there is large variability within the FEP diagnosis in terms of the severity of positive symptoms (from acute to minimal), both because of the effects of antipsychotics on positive symptoms and the existence of different profiles of symptom expression. Additionally, all ARMS patients are experiencing positive symptoms at present by definition (although below the threshold to be categorized as FEP), with a high proportion having comorbidities at baseline (Fusar‐Poli *et al*., 2017), especially anxiety and depressive disorders (Salokangas *et al*., 2012; Svirskis *et al*., 2005). Indeed, it has been suggested that the ARMS concept should be viewed as a syndrome per se that is similar to other psychiatric conditions (such as first episode of psychosis) in terms of distress and impairment (Fusar‐Poli *et al*., 2015) rather than a mere state of risk (McGorry, Hartmann, Spooner, & Nelson, 2018). Finally, it is important to note that ESM ratings of paranoia are not tapping symptoms that meet a diagnostic threshold, but rather capture subjective experiences that range from a subclinical to clinical intensity.

By contrast, groups did not differ in the association of trait ESE and paranoia, highlighting the importance of considering the use of different measures of ESE. Indeed, using different methodologies to assess the same construct might provide distinct, though complementary, information (Carstensen *et al*., 2011; Myin‐Germeys *et al*., 2009). Traditional and real‐life assessments procedures capture different, yet correlated, aspects of subjective experience (Ben‐Zeev, McHugo, Xie, Dobbins, & Young, 2012), probably activating different types of functional *selves* (Conner & Barrett, 2012; Kahneman, 2011; Kahneman & Riis, 2005; Markus & Wurf, 1987). Thereby, measuring ESE in daily‐life taps into a more direct experience (the *experiencing self*), highly influenced by immediate activities and environment, and less biased by cognitive schemas and memory, and reflecting momentary feelings of self‐worth or *state* ESE. By contrast, traditional assessments of ESE tap onto a more reflective and long‐term experience of self‐representation (the *believing self*), evoking *trait* ESE. The magnitude of the correlations between traditional and momentary measures of ESE in our sample, as well as in others (Udachina *et al*., 2009), indicates that they are overlapping but not identical constructs, hinting that state and trait ESE are, indeed, qualitative different phenomena (Brown, Dutton, & Cook, 2001).

To our knowledge, this is the first study that explores the relationship between ISE and momentary paranoia, showing that ISE is not related to momentary paranoia either in ARMS or in FEP patients. This is consistent with a recent meta‐analysis that did not find associations between ISE and paranoia severity in psychosis (Murphy *et al*., 2018). In our sample, ISE was not associated with any of the other measures of self‐esteem (only a small correlation with positive momentary ESE was found). Overall, it seems that ISE and ESE are different phenomena.

Several theoretical implications can be derived from these findings. First, our results do not support Bentall’s ‘defensive model’ of paranoia regarding the putative role of ISE in the development of persecutory delusions. Second, by contrast, it seems that paranoia is more related with the direct expression of negative emotion and ESE, as suggested by Freeman’s multifactorial model of paranoia (Freeman *et al*., 2002). Finally, our findings highlight the relevance of understanding and handling ESE as a construct comprising distinct positive and negative dimensions, which could have relevant clinical implications (Barrowclough *et al*., 2003; Brown *et al*., 1990), and is in line with previous theoretical formulations of ESE (e.g. Andrews, 1998; Owens, 1993).

### Moderation of the association between ESE and Paranoia in daily‐life

As hypothesized, perceived social support moderated the relationship between momentary global ESE and paranoia, consistent with studies showing the protective role of perceived social support in relation to paranoia and positive symptoms (Freeman *et al*., 2011; Lamster, Lincoln, Nittel, Rief, & Mehl, 2017; Norman *et al*., 2005; Sündermann *et al*., 2014). This underscores the relevance of having optimal levels of ESE in combination with positive social environments to mitigate paranoid ideation, as well as the need to address negative interpersonal self‐concepts (Lincoln *et al*., 2010) and exaggerated interpersonal sensitivity (Meisel, Garety, Stahl, & Valmaggia, 2018). Our findings suggest that the interaction of positive emotions with nurturing social environments mitigates paranoid ideation. This pattern in daily‐life confers ecological validity and offers clinical implications for devising resilience‐oriented interventions. These effects were found for both ARMS and FEP groups. By contrast, anxiety amplified the association between poor momentary global ESE and paranoia, in line with the threat anticipation cognitive model of persecutory delusions that attributes a direct role of negative emotions in the genesis and maintenance of paranoia (Freeman, 2007; Freeman *et al*., 2002 **)**, and highlights that dynamic interactions between disturbing emotions drive the development of persecutory delusions.

A different picture appeared when the effects of the moderators were tested separately for positive and negative ESE, highlighting again the importance of separately exploring positive and negative ESE (Barrowclough *et al*., 2003; Stewart *et al*., 2017). Whereas all the moderators had a significant impact on the relationship between positive ESE and paranoia, only feeling cared for by others moderated the association between negative ESE and paranoia, which seems to confer a critical role to negative ESE in relation to paranoia. The nature of the analyses does not allow us to establish causality, but it suggests that negative ESE and paranoia simultaneously serve as cause and consequence of each other (Birchwood, Iqbal, & Upthegrove, 2005; Krabbendam *et al*., 2002; Roe, 2003; Thewissen *et al*., 2008). Interestingly, feeling cared for by others, but not social closeness, dampened the association between negative ESE and paranoia. This is consistent with previous findings that paranoid individuals did not differ in momentary paranoia between familiar and less‐familiar contacts (Collip *et al*., 2011). Furthermore, feeling cared for by others seems to be different in nature from appraisals of social closeness. People can feel close to others because of high familiarity (e.g. in the case of relatives), but that does not entail that they feel cared for by others, which seems to capture a more 'active' protective factor. The social defeat hypothesis argues that 'outsider status', the negative experience of feeling inferior and excluded, acts as common risk factor for schizophrenia (Selten & Cantor‐Graae, 2005) and other psychiatric disorders (Selten, van der Ven, Rutten, & Cantor‐Graae, 2013). We suggest that feeling cared for by others is unequivocally at the opposite end of the cognitive structure of the so‐called 'outsider status' and might buffer the negative experience of social defeat, thus playing a powerful role in decreasing paranoia in real life. This finding should inform the design of personalized interventions in real life using ambulatory assessment methods (Myin‐Germeys *et al*., 2009).

Several limitations of this study must be considered. We used a heterogeneous sample of FEP patients including some individuals with affective disorders with psychotic symptoms (21%). This might affect the direct generalization of these findings to populations restricted to schizophrenia spectrum diagnoses. On average, around 39 per cent of ESM questionnaires were missed by participants due to unknown reasons (e.g. typically participants did not hear the beeping signal, could not attend the questionnaire at that particular time), which might affect the results obtained in this study. However, ESM is an intensive protocol assessment of repeated measures in which missing questionnaires is expected. Finally, we employed a measure of ISE that overcomes some methodological difficulties of previous measures (Nosek & Banaji, 2001); however, the nature of the unconscious self‐related association that the ISE assesses is still unclear (Buhrmester, Blanton, & Swann, 2011). Further research is needed for a better understanding of the measures that seek to delve into the non‐conscious psychological processes.

## Conflict of interest

None.

## Author contribution

Manel Monsonet (Conceptualization; Data curation; Formal analysis; Investigation; Methodology; Validation; Writing – original draft) Thomas R. Kwapil (Data curation; Methodology; Validation; Writing – review & editing) Neus Barrantes‐Vidal, Ph.D. (Conceptualization; Funding acquisition; Project administration; Validation; Writing – review & editing)

## Ethical approval

The authors assert that all procedures contributing to this work comply with the ethical standards of the relevant national and institutional committees on human experimentation and with the Helsinki Declaration of 1975, as revised in 2008.

## Supporting information


**Table S1.** Experience‐Sampling Methodology Questionnaire.

## Data Availability

The data that support the findings of this study are available on request from the corresponding author. The data are not publicly available due to privacy or ethical restrictions.
