# Supplementary material for: Deconstructing the relationships between self‐esteem and paranoia in early psychosis: an experience sampling study
Source: Br J Clin Psychol. 2020 Aug 30;59(4):503–23. doi: 10.1111/bjc.12263 (PMC7693052; doi:10.1111/bjc.12263)
Supplement: Supplementary file 1 — Table S1. Experience‐Sampling Methodology Questionnaire. [file BJC-59-503-s001.docx]

**Supplementary material**

Table S1.

*Experience-Sampling Methodology Questionnaire*

| 1) Right now I feel happy  2) Right now I feel sad  3) Right now I feel anxious (nervous)  4) Right now I fear losing control  5) Right now I feel relaxed 6) Right now I feel angry  7) Right now I feel weird  8) Right now I feel good about myself  9) Right now I feel guilty or ashamed  10) Right now I can concentrate well  11) Right now I have difficulty controlling my thoughts  12) Right now I have no thoughts or emotions  13) Right now my thoughts are strange or unusual  14) Right now I feel tired  15) Right now I feel that others care about me  16) Right now I feel suspicious  17) Right now familiar things have a special meaning or importance to me  18) Right now I do not feel well  19) Right now I feel mistreated  20) I like what I’m doing right now  21) Right now I can do my current activity  22) Right now my sight or hearing seem strange or unusual  23) Since the last beep, I have heard or seen things others could not  24) Right now I feel that someone or something is controlling my thoughts or actions  25) Right now familiar things seem strange and unusual  26) Right now I can cope  27) My current situation is positive  28) My current situation is stressful  29) Since the last beep, I consumed: Food \| Caffeine \| Medication \| Snuff \| Alcohol \| Cannabis or other drugs  30) Right now I am alone Yes = 1, No = 2  If answer is yes to item 30:  31) I am alone because people do not want to be with me  32) Right now I would prefer to be with people  If answer is no to item 30:  33) I feel close to this person (these people)  34) Right now I would prefer to be alone |
| --- |

*Note.* All questions answered from 1 (*not at all*) to 7 (*very much*), unless otherwise noted.
